# Delivery of Peer Support Through a Self-Management mHealth Intervention (Healing Circles) in Patients With Cardiovascular Disease: Protocol for a Randomized Controlled Trial

**DOI:** 10.2196/12322

**Published:** 2019-01-11

**Authors:** Brodie M Sakakibara, Santabhanu Chakrabarti, Andrew Krahn, Martha H Mackay, Tara Sedlak, Joel Singer, David GT Whitehurst, Scott A Lear

**Affiliations:** 1 Faculty of Health Sciences Simon Fraser University Vancouver, BC Canada; 2 Division of Cardiology Providence Health Care Vancouver, BC Canada; 3 Division of Cardiology Vancouver Coastal Health Authority Vancouver, BC Canada; 4 School of Public and Population Health Faculty of Medicine University of British Columbia Vancouver, BC Canada

**Keywords:** cardiovascular disease, eHealth, mhealth, mobile phone, peer support, self-management

## Abstract

**Background:**

Cardiovascular disease (CVD) is a leading cause of hospitalization and death around the world. The prevalence of CVD is increasing and, therefore, development and investigation of effective programs to help people better self-manage their CVD and prevent secondary complications are needed.

**Objective:**

In this paper, we report on a protocol to evaluate Healing Circles—an evidence-based and patient-informed peer support mobile health program designed to facilitate self-management and support patients in their recovery from and management of CVD. We hypothesize that individuals with CVD who use Healing Circles will experience greater improvements to their self-management ability than individuals receiving usual care.

**Methods:**

In this single-blinded (assessor) randomized controlled trial, 250 community-living individuals with CVD will be randomized on a 1:1 basis to either Healing Circles or Usual Care. The primary outcome of self-management will be measured using the Health Education Impact Questionnaire version 3.0. Secondary outcomes include self-efficacy with chronic disease management, health-related quality of life, health resource use and costs, and electronic health literacy. Measurements will be taken at the baseline and every 6 months for 24 months.

**Results:**

The study started recruitment in September 2017. Individuals are currently being recruited for participation, and existing participants are currently on follow-up. Measurements will be taken every 6 months until the study end, which is anticipated in December 2019.

**Conclusions:**

Healing Circles is a novel program aimed toward improving self-management through peer support. Given our real-world study design, our findings will be readily translatable into practice. If the results support our hypothesis, it will indicate that Healing Circles is an effective intervention for improving self-management and reducing health care use.

**Trial Registration:**

ClinicalTrials.gov NCT03159325; https://clinicaltrials.gov/ct2/show/NCT03159325 (Archived by WebCite at http://www.webcitation.org/74DvxVKUd)

**International Registered Report Identifier (IRRID):**

DERR1-10.2196/12322

## Introduction

Cardiovascular disease (CVD) is a leading cause of hospitalization and death around the world [1]. In 2015, 17.7 million people died from a CVD (7.4 million due to coronary artery disease), representing 31% of all deaths worldwide [1]. In the United States, >90 million people are reported to have a CVD, with total direct costs of medical care exceeding US$396 billion in 2012 [2]. Owing to population aging and the rising epidemics of obesity and type 2 diabetes, it is anticipated that the CVD number will continue to grow, along with annual costs that are estimated to reach US$918 billion by 2030 [2].

Patient self-management, defined as an individual’s ability to manage the symptoms, treatment, physical and psychosocial consequences, and lifestyle changes inherent in living with one or more chronic conditions [3], is a cornerstone of treatment for CVD [4]. At present, it is estimated that half of the CVD-related hospital readmissions are due to patients not effectively self-managing their CVD [5,6]. Enhancing patient self-management, therefore, can help prevent patient deterioration and downstream hospitalizations for CVD, as well as reduce health care costs and premature mortality [7-9]. Importantly, patients who adopt self-management practices are empowered with the skills and knowledge necessary to manage their condition for long-term health benefits [7,10]. A key element of effective self-management support includes access to social and peer support networks [10-12]. Patients with CVD who report poor social support have higher readmission rates [13-15] and increased risk of mortality [16-18]. Social support programs that facilitate patients sharing information can reduce depression, provide comfort, restore confidence, improve functional status, and offer practical solutions for self-management [19], which, in turn, can result in lower rates of premature mortality [20] and fewer subsequent CVD events [21].

Despite benefits from self-management programs, questions remain as to how these programs can be implemented. Much discussion has centered on integrating self-management within the health care systems in Canada and the United States [12,22,23]. However, the integration of support for self-management practices into these health care systems has been slow, primarily due to the inherent challenges of systems that have traditionally emphasized the management of acute and episodic conditions rather than the chronic and continuous care patients with CVD require [12,22]. Without the development and testing of accessible and effective self-management programs to help people better manage their CVD and prevent secondary complications, the burden of the disease is likely to reach insurmountable levels.

In this study, we report the protocol of a real-world randomized controlled trial to evaluate Healing Circles—a mobile health (mHealth) self-management and social support program designed to bring peers with CVD together to learn from and support each other in the recovery and daily management of their disease. The primary hypothesis of this study is that individuals with CVD who participate in Healing Circles will experience improvements in their self-management ability, which are significantly greater than individuals receiving usual care. We are also investigating the effects of Healing Circles on the health-related quality of life, self-efficacy with chronic disease management, electronic health (eHealth) literacy, and health care resource use as part of our economic evaluation.

## Methods

### Study Design

**Figure 1:**
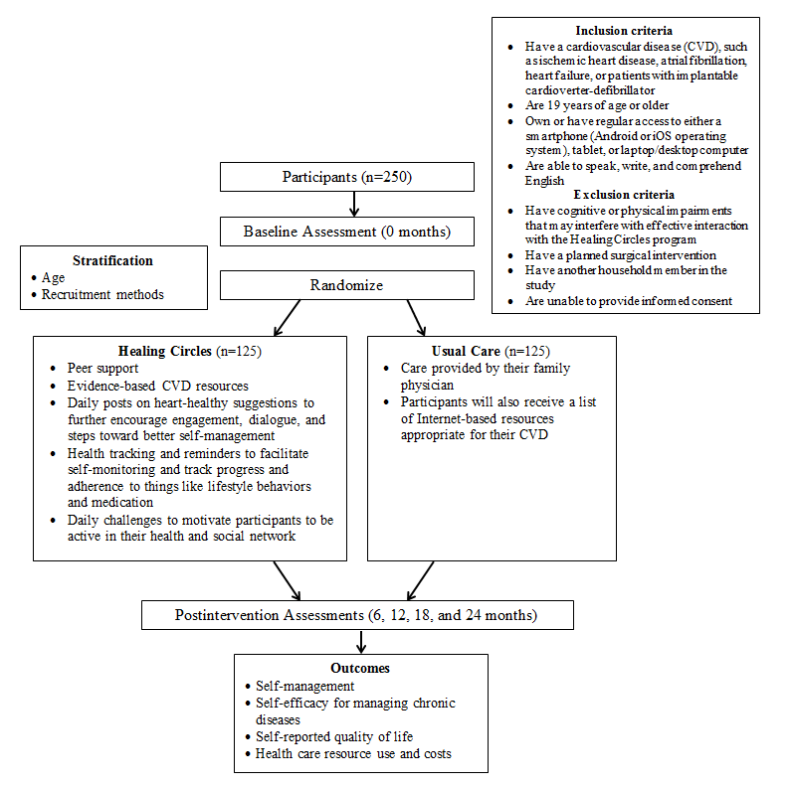
Overview of trial procedures.

This multisite study uses a randomized, controlled, parallel-group, single-blinded (assessor) study design, which has been guided by our Healthcare Innovation Community advisory group of researchers, clinicians, patients, and decision makers. This study has been registered on ClinicalTrials.gov (#NCT03159325). The reporting of this protocol follows the Standard Protocol Items: Recommendations for Intervention Trials [24] guidelines. Figure 1 presents an overview of trial procedures.

### Patient Population

Volunteers from British Columbia, Canada, will be recruited from cardiac outpatient clinics and community-based CVD support groups. Individuals will be included for study if they (1) have a CVD (ischemic heart disease, atrial fibrillation, heart failure, or patients with implantable cardioverter-defibrillator; together these are nearly 80% of patients with CVD); (2) are aged ≥19 years; (3) own or have regular access to either a smartphone (Android or iOS operating system), tablet, or laptop or desktop computer; and (4) can speak, write, and comprehend English. Individuals will be excluded if they (1) have cognitive or physical impairments that may interfere with effective interaction with the Healing Circles program; (2) have a known planned surgical intervention; (3) have another household member in the study; or (4) are unable to provide informed consent.

### Sample Size Estimate

Data from our single-group, pre-post pilot study that examined the proof-of-concept of the Healing Circles program [25] indicated an expected effect size of 0.58 in our primary outcome, the difference in the change in the social integration and support subscale of the Health Education Impact Questionnaire (HeiQ) Version 3 [26]. For this study, we selected a more conservative effect size of 0.33 that is, a mid-to-large effect based on Cohen *f* mid effect (0.25) plus large effect (0.40) divided by 2 [27] to account for comparison between the 2 groups (of note, this estimate may increase slightly in the Usual Care group) and the fact that the parameter estimates from our small pilot study may be highly variable. With an alpha of.05, we calculated a sample size of 198 to have 90% power. To adjust for an expected loss to follow-up rate of ~20%, over the duration of the study, we will recruit a sample size of 250 (125 per group) to retain 198 participants at the study end.

### Randomization and Interventions

After the baseline assessment, participants will be stratified by sex and recruitment site, and randomized using Web-based, computer-generated, random block sizes of 4 and 6 in a 1:1 manner to either the Usual Care or the Healing Circles group. A member of the research team who is not involved with recruitment, assessments, or study intervention will perform the randomization procedures.

#### Usual Care Group

Participants randomized to the usual care group will receive a list of internet-based resources appropriate for their specific CVD. For many participants, usual care will comprise the care provided by their family physician. By virtue of our recruitment, some participants will be attending cardiac outpatient clinics and may receive some self-management education as a result. However, participants are unlikely to have access to formalized peer support programs or regular access to expert input or advice. There will be no contact between the study personnel and Usual Care participants for the study duration or any attempt to influence any aspect of patient care.

#### Experimental (Healing Circles) Group

In addition to receiving care as usual, participants randomized to the intervention group will receive access to and instruction on how to use (ie, provision of an on-boarding document) the Healing Circles program. Healing Circles is a novel, evidence-based, and patient-informed self-management platform designed to support patients with CVD. It uses a private, secure social network that helps connect participants and provides functions to assist participants in self-management through evidence-based principles of behavioral change (eg, social support [11] and self-monitoring [28]).

Healing Circles has 3 following layers: (1) the participant; (2) the participant’s circle, and (3) the broader Healing Circles community. The participant’s circle is the private group that consists of other Healing Circle users that a participant chooses to support him or her (ie, an online support group). These private circles are used for private group or one-on-one chat and communication, peer support, challenges, and sharing. The broader community comprises all Healing Circles users, where participants can see public posts from others not in their circle.

Participants access Healing Circles by downloading or installing, at no cost, the platform onto their personal device (smartphone or tablet). Upon initiating Healing Circles, participants are asked to enter optional information about themselves such as gender, age, cardiac condition, region of residence, types of treatments, and personal interests and hobbies. This information allows the platform to customize and personalize the user experience, from suggesting peers with whom to connect with and add to their support circle, to personalizing content and customizing management tracking tools. However, entering this information is optional and is kept private and secure in accordance with Canadian and American data privacy laws (ie, Personal Information Protection and Electronic Documents Act in Canada and the Health Insurance Portability and Accountability Act in the United States). The participants’ next step is to form their own circle of 8-10 “friends”. Social support is strongest when coming from peers who have similar characteristics and who possess knowledge that is pragmatic and derived from similar lived experiences [19], so Healing Circles offers participants a matching algorithm that can be used to identify similar users to invite to their Circle. The proprietary algorithm is capable of matching from one to several users that help participants develop their Circle. In addition, participants can be invited into other Circles by other users (participants can be in more than one Circle). Similarly, this matching algorithm also matches participants to relevant evidence-based content on Healing Circles, thereby providing a personalized experience. While we will advise participants to use Healing Circles at their own convenience, we will encourage them to create their Circles within 1 week to minimize the time from the baseline assessment and first use. Participants will be instructed to contact the program developers if they experience issues using the program.

Participants also have access to evidence-based educational materials on CVD as well as several functions such as *self-initiated reminders*, which participants can sign themselves up for. These simple reminders are push notifications to prompt participants to enter data. For example, participants can sign up for daily reminders to take their medication and, when prompted, can indicate if they have taken their medication. Furthermore, Healing Circles allows participants to track their progress and adherence to things like lifestyle behaviors and medication (*health tracking*) and to pose *daily challenges* to individuals in their Circle, their entire Circle, or the entire community; these challenges can serve to motivate participants to be active in their health and social network.

### Study Outcome Measures

The primary outcome is the difference in the change in the social integration and support subscale of the HeiQ Version 3 [26] between the Healing Circles and Usual Care groups. HeiQ is a self-management measure developed using Item Response Theory, comprising 8 self-management subscales as follows: health directed activity (4 items); positive and active engagement in life (5 items); emotional distress (6 items); self-monitoring and insight (6 items); constructive attitudes and approaches (5 items); skill and technique acquisition (4 items); social integration and support (5 items); and health service navigation (5 items). Each item is scored using a 4-point response scale (1=strongly disagree; 2=disagree; 3=agree; 4=strongly agree). A mean score is derived for each subscale with higher scores indicating better self-management ability, with the exception of the emotional well-being subscale where a higher mean score is indicative of lower self-management ability.

We selected the 5-item social integration and support subscale as our primary outcome because the items align closely with the Healing Circles intervention, and results from our pilot study suggested that this subscale is most responsive to changes as a result of the Healing Circles program [25]. Moreover, this subscale has been shown to have high internal consistency reliability (Cronbach alpha=.86) [26].

Secondary outcomes include the 7 other subscales within the HeiQ as well as several other variables as follows. *Self-efficacy* will be measured using the 6-item Self-efficacy for Managing Chronic Disease Scale [29]. This 6-item scale covers several domains that are common across many chronic diseases, including symptom control, role functioning, emotional functioning, and communicating with physicians and has high internal consistency reliability (Cronbach alpha=.91) [29]. Responses for each item range from 1 (not at all confident) to 10 (totally confident). Higher mean scores indicate higher self-efficacy.

Generic preference-based measures of *health-related quality of life* and *capability well-being* will be collected using the EuroQoL-5 dimension 5-level version (EQ-5D-5L) [30] and ICEpop CAPability measure for Adults (ICECAP-A) [31], respectively. These two measures will be used in the economic analysis (see below). The EQ-5D-5L is a widely used outcome measure for the estimation of quality-adjusted life years (QALYs). Developed by the EuroQol Group, the EQ-5D-5L was introduced to improve the sensitivity as compared with the EQ-5D 3-level version [32]. The ICECAP-A is based on Amartya Sen’s capability approach, which distinguishes between capabilities and achieved functioning [31]. It accounts for the fact that a person’s capabilities (what a person can do) may differ from their functioning (what a person actually does), and goes beyond health-related aspects of the quality of life. Both EQ-5D-5L and ICECAP-A have been validated in a range of clinical contexts [33-36].

*Health care resource use* data will be collected from a combination of medical records and self-report questionnaires. The self-report questionnaires will be variants of versions that have been used in previous trial-based economic evaluations [37-39]. Data collection will focus on key cost drivers, such as hospital attendances (inpatient stays, outpatient appointments, and any other hospital visits to health care practitioners), consultations with primary health care providers (eg, family doctors and nurse practitioners), and prescribed medications. Details of nonhealth care costs will be collected, including out-of-pocket expenses, periods of work absence, as well as an assessment of the use of other mHealth -based applications. The dollar value of all resources will be estimated from a number of sources, including local health authority accounts data, Canadian Institute for Health Information patient cost estimator for acute sector admissions [40], and self-reported costs.

*Usage of Healing Circles* will be assessed by tracking the frequency of using Healing Circles features (eg, log-ins and the frequency of use of various platform features) using program metrics. Furthermore, we will assess what devices participants used to access the platform, as well as engagement and communication with other Healing Circles users.

Finally, *participants’ perceptions, attitudes, and satisfaction* with Healing Circles will be assessed using qualitative interview methodologies at the end of the study. A subgroup of 20-30 participants in the Healing Circles group will be recruited and taken through semistructured, open-ended interviews to explore their experiences and the factors that influence acceptance and the use of the Healing Circles program. Potential interview participants will be identified by stratified purposeful sampling, with representation from both sexes, type of CVD, urban or rural residence, and highly active and less-active Circles. A 3-stage analysis of interview transcripts (open-coding, axial coding, and selective coding) will be guided by an inductive iterative approach [41,42]. The interviews will be conducted by phone and recorded and transcribed with personal identifiers removed.

### Statistical Analyses

Descriptive statistics will be used to characterize the sample. For the analysis of the primary outcome, the social integration, and support subscale of the HeiQ, we will evaluate the difference in change scores between groups using a mixed-effects model that takes into account the repeated measures of the outcome. The model will incorporate the strata of sex and method of recruitment, as well as control for sociodemographic covariates such as age and sex.

With the exception of the data collected for the economic analysis, all other continuous outcomes (our secondary outcomes), including the other 7 subscales of the HeiQ, will also be assessed using a mixed-effects model. In addition, we will investigate whether there are associations of the primary and secondary outcomes with sex, urban or rural residence, disease status, and eHealth literacy. Nonnormally distributed continuous variables will be transformed prior to analyses. All analyses will be tested using an alpha of.05.

### Economic Analyses

The primary framework for the economic analysis will be a cost-consequence analysis, where resource use, costs, and outcomes of the 2 treatment groups are listed separately in a disaggregated format (eg, hospital costs, out-of-pocket expenses, self-management outcomes, health-related quality of life, and capability well-being). The time horizon for the cost-consequence analysis will complement the study’s design for the follow-up to maximize the use of available data (ie, 0-6, 0-12, 0-18, and 0-24 months).

We will also perform a cost-utility analysis from the perspective of the publicly funded health care payer, in line with recommendations from the Canadian Agency for Drugs and Technologies in Health [43]. Health outcomes will be expressed as QALYs, with QALY estimates generated from EQ-5D-5L responses using a Canadian value set [44]. The time horizon for the cost-utility analysis will be dependent on the sample sizes observed at 12-, 18-, and 24-month follow-up. In addition, differences between groups in costs and QALYs will be expressed using the cost-per-QALY ratio, which provides an estimate of the additional cost required to gain each additional unit of outcome.

## Results

### Assessments

#### Baseline

Baseline will consist of the collection of our primary and secondary outcomes that require pre and post measurements, as well as social demographic data, medical history, and eHealth literacy using the eHealth Literacy Scale (eHEALS) [45], to assess the participants’ use of technology to access and evaluate health information. The eHEALS is used to assess the combined knowledge, comfort, and perceived skills at finding, evaluating, and applying electronic health information to health problems.

#### Follow-Up

Every 6 months between randomization and the study’s end (anticipated in December 2019), participants will undergo an assessment of outcomes only. Those recruited at the end and beginning of recruitment will experience follow-up periods ranging from 6 to 24 months (2 years), respectively. We expect an average of 18 months of follow-up. In addition, we anticipate that the main effects on self-management will occur early after randomization; however, extended follow-up will allow us to investigate the sustainability of any observed effect, Healing Circles’ use over time, and health care use for an extended period. Participants may complete data collection either in-person with a blinded assessor, self-report through mailed responses, or on the Web. The study started recruitment in September 2017. Individuals are still currently being recruited for participation, and existing participants are currently on follow-up.

### Study Organization and Funding

This study is funded by the Canadian Institutes of Health Research eHealth Innovations Partnership Program and the Michael Smith Foundation for Health Research. Ethical approval has been obtained, all study staff have been hired and trained, and recruitment is currently under way.

## Discussion

Healing Circles is a novel program aimed at improving self-management through peer support, as well as recovery from and management of CVD. Given our real-world study design and the participatory aspects of engaging end users, our findings could be readily translated into practice. If the results support our hypotheses, it will indicate that Healing Circles is an effective intervention for improving self-management and reducing health care use. If so, this robust assessment of the implementation and use of Healing Circles could be used to inform future design of the program and inform plans for scale-up.

## Appendix

Multimedia Appendix 1 Peer-reviewer report from the Canadian Institutes of Health Research.

## Abbreviations

CVD: cardiovascular disease
eHealth: electronic health
EQ-5D-5L: EuroQoL-5 dimension 5-level version
HeiQ: Health Education Impact Questionnaire
ICECAP-A: ICEpop CAPability measure for Adults
mHealth: mobile health
QALY: quality-adjusted life year
