# Supplementary material for: Delivery of Peer Support Through a Self-Management mHealth Intervention (Healing Circles) in Patients With Cardiovascular Disease: Protocol for a Randomized Controlled Trial
Source: JMIR Res Protoc. 2019 Jan 11;8(1):e12322. doi: 10.2196/12322 (PMC6330197; doi:10.2196/12322)
Supplement: Multimedia Appendix 1 [file resprot_v8i1e12322_app1.pdf]

Review Type/Type d'évaluation: Committee Member 1/Membre de comité 1
Name of Applicant/Nom du chercheur: LEAR, Scott A
Application No./Numéro de demande: 348469
Agency/Agence: CIHR/IRSC
Competition/Concours: 2015-06-03 Operating Grant: eHealth Innovations Initiative: eHealth Innovation Partnership Program (eHIPP)/Subvention de fonctionnement: Initiative Innovations en cybersanté : Programme de partenariats pour l’innovation en cybersanté (PPIC)
Committee/Comité: eHealth Innovation Partnership Program (eHIPP): Support of Seniors/Programme de partenairiats pour l'innovation en cybersanté-L'appui awx personnes âgéess
Title/Titre: Delivery of self-management through a peer-support telehealth intervention in patients with cardiovascular disease: The Healing Circles Project

---

Assessment/Évaluation:

Brief synopsis

This project proposes to test the effectiveness of the use of the Healing Circles, peer support and social networking platform, to improve self-management skills (e.g., adherence to medication and healthy life style) for older patients with CVD who tend to be isolated, for cardiovascular disease (ischemic heart disease, arrhythmias and HF) QOL, and health service use. They will test using 250 participants who are randomly assigned to the intervention group and usual care. 8-10 matched participants form a group and receive information/education from the Heart and Stroke Foundation of Canada (experts). Technology was developed by Curio (Vancouver based) and delivers a low-cost and highly accessible solution through PC, tables, and smart phones, thereby readily scalable. Nurses in clinics will introduce patients to the system. Due to minimal involvement with a current health care system, the scalability has great potential. For the RCT, the intervention is 6 months and will follow up to 2.5 years.

Comments

This is an interesting and unique proposal that does not involve clinical settings to improve patients’ health behaviors through peer support, although can be controversial. This has an advantage for patients due to no cost and may disseminate fast and can engage any stage of illness conditions. Include objective measurements for health behaviors are lacking.

Planned Intervention:

Strength

1. This proposal deals with health behavior promotion necessary for chronic disease management outside the clinical network.
2. This operation is free for enrollees and they can invite a person to be in a group depending on their profile and can be in more than one group. Since this acts like social media, older people may really use it. Expert support, reminders, health tracking and challenges, as well as technical support are helpful.
3. The scalability is good due to very minimal involvement in the current health care system, and involvement of the Heart and Stroke Foundation (HSF) as well as the Canadian Cardiovascular Society.
4. This is a great way for the HSF to disseminate knowledge.

5. **Weakness**

**Review Type/Type d'évaluation:**	Committee Member 1/Membre de comité 1
**Name of Applicant/Nom du chercheur:**	LEAR, Scott A
**Application No./Numéro de demande:**	348469
**Agency/Agence:**	CIHR/IRSC
**Competition/Concours:**	2015-06-03 Operating Grant: eHealth Innovations Initiative: eHealth Innovation Partnership Program (eHIPP)/Subvention de fonctionnement: Initiative Innovations en cybersanté : Programme de partenariats pour l'innovation en cybersanté (PPIC)
**Committee/Comité:**	eHealth Innovation Partnership Program (eHIPP): Support of Seniors/Programme de partenairiats pour l'innovation en cybersanté-L'appui awx personnes âgéess
**Title/Titre:**	Delivery of self-management through a peer-support telehealth intervention in patients with cardiovascular disease: The Healing Circles Project

Assessment/Évaluation:

1. What severity of CVD will form a group? Is it helpful if their severity varies? Related to that, who among CVD patients are targeted is not clear. They should have passed critical phase after ED or hospital discharge?
2. If a patient becomes a negative effect for other group members, how would this situation be handled? This may come up when they do the actual study.
3. For scalability, who will be responsible for matching and how will the catching coordinator be funded?

eHealth Innovation Evaluation:

Strength

1. **PI is well qualified: Chair in Cardiovascular Prevention Research in a hospital and the PI of the BC Alliance on Telehealth Policy and Research. Others are qualified.**

Weakness

1. This is not a weakness but on page 8, in Inclusion criteria, iPhone is omitted but this was used for the pilot study, so it should be included.
2. How medication adherence will be measured? There is no objective measure for health behaviors.
3. Is this an intent-to-treat analysis?
4. Because of non-involvement of clinical settings and specific devices, and rather simple technology, which was tested and apparently further improvement is not needed, the study can be done in 3 years.
5. Who will train/teach technology to participants? A Study coordinator? How the involvement of study coordinator will affect scalability of this project?

Team:

Strength

**Review Type/Type d'évaluation:**	Committee Member 1/Membre de comité 1
**Name of Applicant/Nom du chercheur:**	LEAR, Scott A
**Application No./Numéro de demande:**	348469
**Agency/Agence:**	CIHR/IRSC
**Competition/Concours:**	2015-06-03 Operating Grant: eHealth Innovations Initiative: eHealth Innovation Partnership Program (eHIPP)/Subvention de fonctionnement: Initiative Innovations en cybersanté : Programme de partenariats pour l'innovation en cybersanté (PPIC)
**Committee/Comité:**	eHealth Innovation Partnership Program (eHIPP): Support of Seniors/Programme de partenairiats pour l'innovation en cybersanté-L'appui awx personnes âgéess
**Title/Titre:**	Delivery of self-management through a peer-support telehealth intervention in patients with cardiovascular disease: The Healing Circles Project

Assessment/Évaluation:

1. Involvement of the Heart and Stroke Foundation of Canada and the Canadian Cardiovascular Society is strength.
2. The team and technology (Curatio) is very strong evidenced by a successful history and track record related to eHealth.

Weakness

1. A biostatistician should be in the team, separate from a health economist.

Budget: This is not a budget but a timeline.This study can be done in 3 years.
Sex and/or gender consideration: Satisfied

**Review Type/Type d'évaluation:**	Committee Member 2/Membre de comité 2
**Name of Applicant/Nom du chercheur:**	LEAR, Scott A
**Application No./Numéro de demande:**	348469
**Agency/Agence:**	CIHR/IRSC
**Competition/Concours:**	2015-06-03 Operating Grant: eHealth Innovations Initiative: eHealth Innovation Partnership Program (eHIPP)/Subvention de fonctionnement: Initiative Innovations en cybersanté : Programme de partenariats pour l'innovation en cybersanté (PPIC)
**Committee/Comité:**	eHealth Innovation Partnership Program (eHIPP): Support of Seniors/Programme de partenairiats pour l'innovation en cybersanté-L'appui awx personnes âgéess
**Title/Titre:**	Delivery of self-management through a peer-support telehealth intervention in patients with cardiovascular disease: The Healing Circles Project

Assessment/Évaluation:

review pending

Review Type/Type d'évaluation: Committee Member 3/Membre de comité 3
Name of Applicant/Nom du chercheur: LEAR, Scott A
Application No./Numéro de demande: 348469
Agency/Agence: CIHR/IRSC
Competition/Concours: 2015-06-03 Operating Grant: eHealth Innovations Initiative: eHealth Innovation Partnership Program (eHIPP)/Subvention de fonctionnement: Initiative Innovations en cybersanté : Programme de partenariats pour l’innovation en cybersanté (PPIC)
Committee/Comité: eHealth Innovation Partnership Program (eHIPP): Support of Seniors/Programme de partenairiats pour l'innovation en cybersanté-L'appui awx personnes âgéess
Title/Titre: Delivery of self-management through a peer-support telehealth intervention in patients with cardiovascular disease: The Healing Circles Project

---

Assessment/Évaluation:

Planned Intervention:
This intervention is very relevant to the senior population with cardiovascular disease – a large group within the Canadian healthcare system. The technology has been piloted with success and will reach TRL level 7 within first year of project. Providing a web-based platform that involves social support is innovative and cost effective. Scalability on proof of concept is very feasible. The ‘stories’ of patients with CVD strengthen the patient-oriented approach. With the strong support of the HSF, end-user co-development will more likely be effective. The implementation plan is very dependent on CVD patient comfort with the social networking concept and willingness to form their primary circle of support which could be a drawback. Clinician support will be dependent on the time it takes to monitor their patients using social media.
eHealth Innovation Evaluation:
The sample size is quite small but appears to reflect the local population who currently have access to the platform technology. The outcomes: facilitated self-management with peer support, are ones that are advocated for by other patient groups, in particular seniors, who want to maintain control of their own health. Greater patient engagement in the analysis will lead to a wider audience for KT spread. The current environment determined by the level of acceptance of mobile technologies in our society is very good for this innovation.
Team:
The partnership between the health-care innovation community and the health technology partners seems quite strong given that they have engaged in previous partnerships. The support of the Heart and Stroke Foundation also speaks to a strong team ready to co-develop and integrate this eHealth solution. The scientific team includes leaders from academia, clinical areas, and the HSF which has the capacity to conduct the proposed activities and undertake the evaluation.

Review Type/Type d'évaluation: Committee Member 4/Membre de comité 4
Name of Applicant/Nom du chercheur: LEAR, Scott A
Application No./Numéro de demande: 348469
Agency/Agence: CIHR/IRSC
Competition/Concours: 2015-06-03 Operating Grant: eHealth Innovations Initiative: eHealth Innovation Partnership Program (eHIPP)/Subvention de fonctionnement: Initiative Innovations en cybersanté : Programme de partenariats pour l'innovation en cybersanté (PPIC)
Committee/Comité: eHealth Innovation Partnership Program (eHIPP): Support of Seniors/Programme de partenairiats pour l'innovation en cybersanté-L'appui awx personnes âgéess
Title/Titre: Delivery of self-management through a peer-support telehealth intervention in patients with cardiovascular disease: The Healing Circles Project

---

Assessment/Évaluation:

Cardiovascular disease is a leading cause of death and disability in Canada costing $22.2 billion annually. Patient self-management is key in better patient outcomes and reducing hospital visits. This project plans to build the popularity of social networks and create mini-health circles and groups to facilitate support from other patients.

The population is relevant and has appropriate challenges; the gap is in the effective delivery of self-managed support. Effective delivery can reduce depression, provide comfort, restore confidence, improve functional status and offer practical solutions resulting reduced mortality and subsequent hospital visits. The technology appears to be a TRL 7 and can easily move higher based on its current state. Based on provided testimonials, previous testing and partnerships and overall plan it should improve outcomes for the targeted population with respect to self-management. The solution itself is cloud based and easily scalable. Given its social nature it would most likely benefit from scaling and having more users involved. Once it is proven and environmental and use-case is sorted out, scaling is relatively easy. The environment is hospital base and under a health research team.

The partners for this project have worked together previously. They have developed the preliminary and early testing portions of this project already and have done some testing and implementation to get it to where it is as right now. Based on this it is believed there is a high probability that it will succeed. The technology platform is itself ready, it would be relatively minimal work to scale up the solution and get it deployed based on the previous work already completed and should have ample capacity to deliver the solution. All parties appear to have high quality members with relevant experience necessary and the ability to make the decisions required and the technology partner has a strong advisory group. The health research team and hospital setting are suitable, allow for direct access to the necessary people and the team has worked together on this project before with success. The people involved are active and accessible and the location and infrastructure are available and have been developed and setup previously.

The technology company is small and young however the major financial requirements have already been fulfilled and the additional requirements for the purposes of this application are minimal and can satisfied. The technology platform benefit is one that it facilitates and allows patients to support each other through

**Review Type/Type d'évaluation:**	Committee Member 4/Membre de comité 4
**Name of Applicant/Nom du chercheur:**	LEAR, Scott A
**Application No./Numéro de demande:**	348469
**Agency/Agence:**	CIHR/IRSC
**Competition/Concours:**	2015-06-03 Operating Grant: eHealth Innovations Initiative: eHealth Innovation Partnership Program (eHIPP)/Subvention de fonctionnement: Initiative Innovations en cybersanté : Programme de partenariats pour l’innovation en cybersanté (PPIC)
**Committee/Comité:**	eHealth Innovation Partnership Program (eHIPP): Support of Seniors/Programme de partenairiats pour l'innovation en cybersanté-L'appui awx personnes âgéess
**Title/Titre:**	Delivery of self-management through a peer-support telehealth intervention in patients with cardiovascular disease: The Healing Circles Project

Assessment/Évaluation:

recovery process thus improving the patient outcomes and possibly lowering the amount of visits and interactions with medical professionals in a hospital environment. The commercialization potential of the technology is strong as it is already selling and being implemented in some forms. This application would allow the technology to be finalized for this particular demographic and be scaled up. The infrastructure currently exists for the platform which is already operational on various levels so the required commercialization is within the capacity of the company. The previous work demonstrates the customer market and as a niche it should be highly adoptable. The main barrier will be the business model and backing up the sales pitch with hard data proving this works and will save money vs being a perk for a potential client. The budget itself appears appropriate, not excessive and meets the requirements and this project should be funded.

Review Type/Type d'évaluation: SO Notes /Notes de l'agent scientifique
Name of Applicant/Nom du chercheur: LEAR, Scott A
Application No./Numéro de demande: 348469
Agency/Agence: CIHR/IRSC
Competition/Concours: 2015-06-03 Operating Grant: eHealth Innovations Initiative: eHealth Innovation Partnership Program (eHIPP)/Subvention de fonctionnement: Initiative Innovations en cybersanté : Programme de partenariats pour l'innovation en cybersanté (PPIC)
Committee/Comité: eHealth Innovation Partnership Program (eHIPP): Support of Seniors/Programme de partenairiats pour l'innovation en cybersanté-L'appui awx personnes âgéess
Title/Titre: Delivery of self-management through a peer-support telehealth intervention in patients with cardiovascular disease: The Healing Circles Project

Assessment/Évaluation:

Application Number: 348469

Title: Delivery of self management through a peer support telehealth intervention in patients with cardiovascular disease. The healing circles project.

This proposal nicely supports self management for people with cardiovascular disease using peer support via a web based platform. The proposal is innovative. The technology readiness is strong. The technology will match together peers for self management support. This proposal is an extension of a 10 week pilot study. Participant feedback from the pilot study was positive. This is a person centred, non-paternalistic strategy. Partnerships are strong and well established. The team has co-developed the design with the patients across Canada. There is good diversity of human resources (i.e. heart and stroke, ask an expert, peers). Confidentiality is addressed by using an online pseudonym. Protection from online bullying was addressed.

More information is needed to understand medication adherence strategies.
